# Beyond SARS-CoV-2: epidemiological surveillance of respiratory viruses in Jalisco, Mexico

**DOI:** 10.3389/fpubh.2023.1292614

**Published:** 2024-01-11

**Authors:** Isaac Murisi Pedroza-Uribe, Natali Vega Magaña, José Francisco Muñoz-Valle, Marcela Peña-Rodriguez, Ahtziri Socorro Carranza-Aranda, Rocío Sánchez-Sánchez, Alberto Anthony Venancio-Landeros, Octavio Patricio García-González, Jacob Jecsan Zavala-Mejía, Moisés Ramos-Solano, Oliver Viera-Segura, Mariel García-Chagollán

**Affiliations:** ^1^Doctorado en Microbiología Médica, Centro Universitario de Ciencias de la Salud, Universidad de Guadalajara, Guadalajara, Mexico; ^2^Laboratorio de Diagnóstico de Enfermedades Emergentes y Reemergentes (LaDEER), Centro Universitario de Ciencias de la Salud, Universidad de Guadalajara, Guadalajara, Mexico; ^3^Instituto de Investigación en Ciencias Biomédicas, Centro Universitario de Ciencias de la Salud, Universidad de Guadalajara, Guadalajara, Mexico; ^4^Doctorado en Ciencias Biomédicas, Centro Universitario de Ciencias de la Salud, Universidad de Guadalajara, Guadalajara, Mexico; ^5^Instituto Traslacional de Singularidad Genómica (ITRASIG), Irapuato, Mexico; ^6^Licenciatura en Médico Cirujano y Partero, Centro Universitario de Ciencias de la Salud, Universidad de Guadalajara, Guadalajara, Mexico; ^7^Instituto de Investigación en Cáncer en la Infancia y Adolescencia (INICIA), Centro Universitario de Ciencias de la Salud, Universidad de Guadalajara, Guadalajara, Mexico

**Keywords:** SARS-CoV-2, respiratory viruses, epidemiological survey, clinical impact, viral coinfections

## Abstract

**Introduction:**

Respiratory viral infections represent a significant global health burden. Historically, influenza, rhinovirus, respiratory syncytial virus, and adenovirus have been the prevalent viruses; however, the landscape shifted with the widespread emergence of SARS-CoV-2. The aim of this study is to present a comprehensive epidemiological analysis of viral respiratory infections in Jalisco, Mexico.

**Methods:**

Data encompassing individuals with flu-like symptoms from July 2021 to February 2023 was scrutinized for viral diagnosis through PCR multiplex. The effect of social mobility on the increase in respiratory viral diagnosis infection was considered to estimate its impact. Additionally, sequences of respiratory viruses stored in public databases were retrieved to ascertain the phylogenetic classification of previously reported viruses in Mexico.

**Results:**

SARS-CoV-2 was the most detected virus (*n* = 5,703; 92.2%), followed by influenza (*n* = 479; 7.78%). These viruses were also found as the most common co-infection (*n* = 11; 50%), and for those with influenza, a higher incidence of severe disease was reported (*n* = 122; 90.4%; *p* < 0.001). Regarding comorbidities and unhealthy habits, smoking was found to be a risk factor for influenza infection but a protective factor for SARS-CoV-2 (OR = 2.62; IC 95%: 1.66–4.13; OR = 0.65; IC 95%: 0.45–0.94), respectively. Furthermore, our findings revealed a direct correlation between mobility and the prevalence of influenza infection (0.214; *p* < 0.001).

**Discussion:**

The study presents evidence of respiratory virus reemergence and prevalence during the social reactivation, facilitating future preventive measures.

## 1 Introduction

Respiratory infections are considered nowadays a significant global health burden, causing a wide range of respiratory tract infections, mainly in the upper respiratory tract (1). Before the COVID-19 pandemic, influenza, rhinovirus, respiratory syncytial virus, and adenovirus were considered the most common etiological agents of respiratory disease (2). Except for influenza, these viruses were referred to only as “common colds,” and only a few studies focused on their epidemiological and clinical characterization (2).

Since the emergence of SARS-CoV-2 in 2019, countries have conducted strategies for viral control; one of the most significant was the non-pharmaceutical interventions consisting of city lockdowns, physical distancing, use of personal protective equipment, and individual hygiene practices (3). For instance, there has been a decrease in reported cases of the syncytial virus, Influenza A and B virus, parainfluenza virus 1–3, adenovirus, and human metapneumovirus from 2011 to 2022 (4, 5). Overall, there was a significant decline in seasonal influenza reports during the COVID-19 pandemic in many countries, including the United States, Japan, England, Australia, Canada, South Africa, Singapore, Taiwan, South Korea, and Chile (4, 6, 7). Nevertheless, in 2021, the National Health Institute reported a reemergence of non-SARS-CoV-2 respiratory viruses in the respective countries (4, 8). The viral epidemiological fluctuation could be attributable to the elimination of the non-pharmaceutical interventions and a possible replication interference between viruses (4, 9).

The clinical presentation of respiratory viral infections can vary widely, ranging from asymptomatic or mild illness to severe respiratory distress and organ failure. Coinfections with multiple respiratory viruses are uncommon, and whether they can complicate the clinical course and management of affected individuals is not well described. Moreover, underlying comorbidities, such as diabetes and hypertension, or unhealthy habits, such as smoking, can increase the risk of severe illness and complications (2, 10).

Surveillance systems play a critical role in monitoring the circulation and genetic evolution of respiratory viruses, providing essential data for public health responses, and aiding in the development of targeted interventions. According to the PAHO report, respiratory viruses followed a common epidemiological pattern in Latin America until 2020, with the emergence of the SARS-CoV-2 pandemic, in which there was a decline in the number of cases detected; but, in 2022, an increase in the diagnosis of non-SARS-CoV-2 respiratory viruses was reported (11). In Mexico, the thoroughness of the epidemiological description of SARS-CoV-2 differs from other respiratory viruses; even for the influenza virus, diagnosis and reports are uncommon. The national health institution in Mexico reported more than 10 million acute respiratory infections in 2022 (12), yet these were categorized collectively as acute respiratory infections without viral classification; Jalisco state fits in this same scenario, with no viral respiratory characterization carried despite the augment of the diagnosis of non-SARS-CoV-2 infection (12). Moreover, a previous study described the circulation of a high diversity of respiratory viruses (13). This work aims to provide an overview of the diversity of respiratory viruses and discuss the epidemiology, clinical manifestations, and social impact on the transmission dynamics, as well as its implications on disease severity.

## 2 Materials and methods

### 2.1 Study population and sample processing

As part of a comprehensive strategy to study and monitor the epidemiology of respiratory viruses in the Jalisco state, the Universidad de Guadalajara established a diagnosis laboratory during the COVID-19 pandemic for outpatient SARS-CoV-2 epidemiological surveillance. In this context, nasopharyngeal samples and clinical-demographic data from patients with flu-like illness (ILI) and severe acute respiratory infections (SARI) were retrospectively recovered. The eligibility criteria for the patients enrolled in the present study were as follows: (i) outpatient of any age or sex group requiring molecular diagnosis at the Laboratorio de Diagnóstico de Enfermedades Emergentes y Reemergentes (LaDEER; laboratory validated by the Mexico National Health Institute for respiratory viral diagnosis for epidemiological surveillance); (ii) people who had three or more ILI or SARI symptoms, according to the WHO surveillance case definitions, or other symptoms related to respiratory infection such as fever ≥37.0°C, anosmia, dysgeusia, cough, nasal congestion, chest pain, headache, among others. For the purpose of this study, severity degree was defined according to the number of symptoms; (iii) individuals previously in contact with people with a respiratory infection. All the clinical and epidemiological information, such as comorbidities, symptoms, and demographics, was retrieved by implementing a telephone survey, as mentioned previously (14).

A total of 6,184 nasopharyngeal samples were processed by RT-qPCR. The viral RNA was extracted with the Viral RNA Auto Extraction & Purification Kit (Cat. 3103010059, 3DMed) using the ANDiS 350 Automated Nucleic Acid Extraction System (3DMed). Initially, all samples were examined the same day they were sampled for the most common respiratory viruses, influenza, and SARS-CoV-2, employing the COVIFLU Kit Multiplex (Cat. G2LCoFM-04, Genes2life SAPI de CV, Irapuato, Mexico), which identifies the N gene of SARS-CoV-2, and the coding region of matrix protein (M) for influenza A and B using a Quant Studio 5 (Applied Biosystems); after this analysis, RNA and oro-nasopharyngeal tube were stored at −80°C for subsequent examinations. The samples that were negative for SARS-CoV-2 were screened for other viral infections using Bluefinder 22 (Cat. G2LBF22–01, Genes2Life, Mexico) in the IntelliQube automated PCR instrument (BioSearch Technologies), according to the manufacturer’s instructions; this kit is designed for the diagnosis of 22 respiratory pathogens, such as Rhinovirus, Enterovirus, Bocavirus, Metapneumovirus, Adenovirus, Influenza H1N1 (09 pdm), Influenza H3N2, Influenza B (Victoria and Yamagata lineages), Syncytial A/B, SARS-CoV-2, Parainfluenza (1, 2, 3, and 4), and Human coronavirus (OC43, 229E, NL63, and HKU1). A sample was considered positive when Ct-values were below 35 and when a clear sigmoid curve was observed for the corresponding marker; additionally, a coinfection was defined when an individual tested positive for two or more viruses.

### 2.2 Mobility data report retrieval

In order to evaluate the relationship between the epidemic behavior of respiratory pathogens and the mobility rate in the Jalisco state, we used the database from Google, LLC website (15). With this information and the epidemiological data, we analyzed the shifting patterns of respiratory virus distribution according to the impact of the local population mobility in the Jalisco state.

### 2.3 Phylogenetic analysis from Mexico

A comprehensive search through the Nucleotide section in the public database from the National Center for Biotechnological Information (NCBI), using the keywords “(Virus),” “and,” “Jalisco,” “complete genome” of the main respiratory virus circulating in West Mexico was performed; however, since no results were obtained, we amplified the search to include the entirety of the country. A search for Influenza, Respiratory Syncytial Virus, Human Coronavirus, Adenovirus, Parainfluenza, and Rhinovirus was carried out. Using only the downloaded sequence data, we performed a multiple alignment using MAFFT v.7. A phylogenetic tree was constructed using the Neighbor-Joining algorithm, employing Tamura-Nei as the substitution model of DNA evolution. Phylogenetic tree statistical reliability was evaluated by bootstrap analysis of 10,000 replicates.

### 2.4 Statistical analysis

All qualitative data was summarized as frequencies and percentages, while quantitative data as median and standard deviation. The chi-square test or Fisher’s exact test was performed for comparative analyses. Kruskal-Wallis and Nemenyi *post hoc* tests were used to determine the most common combinations of symptoms and the most prevalent comorbidities. In addition, the correlation between the mobility rate from July 2021 to February 2023 with viral incidence was evaluated by the Kendall-Tau test. All data was analyzed with R Studio software (RStudio Team, 2020) and graphics in GraphPad Prism version 9. Statistically significant differences were considered with a cut-off value of *p* <0.05.

## 3 Results

### 3.1 Demographic and clinical data

A total of 6,184 individuals were included in the present study, corresponding to 24.1, 75.8, and 0.1% recruited in 2021, 2022, and 2023, respectively. The overall mean age of the population was 37.29 ± 15.98 (males 37.79 ± 16.33; females 36.91 ± 15.69), with a predominant representation of female individuals (*n* = 3,533, 57.1%). Data stratification involved categorizing participants into age groups (<18, 18–60, and > 60 years) to compare the distribution between males and females. The main prevalence was observed in the 18–60 age group, consisting of 4,947 individuals (80%), with a female predominance. 3,591 individuals (70.2%) of the population manifested symptoms; headache was reported by 46.6% (*n* = 2,421; from 5,190 with clinical data) of the individuals, followed by cough and sore throat with 42.1% (*n* = 2,183) and 39.2% (*n* = 2,032), respectively. Moreover, the comparative analysis revealed a higher proportion of females experiencing symptomatology compared to males and, excluding fever, fatigue, myalgia, and arthralgia, common respiratory symptoms were more common in women.

Furthermore, the survey revealed comorbidities or unhealthy habits in 26.4% (*n* = 1,372) of the patients, with obesity as the most prevalent condition (10.8%; *n* = 559), followed by smoking and hypertension (9.8 and 6.3%, respectively). Additionally, males presented the highest proportion of any comorbidity. When evaluating comorbidity information independently, the proportion of males reporting a previous disease was the largest (Table 1).

**Table 1:** Clinical and demographic information of the included population.

Variable	Total n (%)	Male n (%)	Female n (%)	P-significance
	6,184	2,651 (42.9)	3,533 (57.1)	<0.001
Age	37.29 ± 15.98	37.79 ± 16.33	36.91 ± 15.69	0.046
Age group (years)				0.007
<18	591 (9.6)	278 (10.5)	313 (8.9)	NS
18–60	4,947 (80)	2072 (78.2)	2,875 (81.4)	<0.001
>60	646 (10.4)	301 (11.4)	345 (9.8)	NS
Comorbidities (n)		n (%)		
Populations manifesting comorbidities	1,372 (26.4)	635 (28.8)	737 (24.7)	<0.001
Diabetes	221 (4.3)	107 (4.9)	114 (3.8)	NS
Obesity	559 (10.8)	247 (11.2)	312 (10.4)	NS
Hypertension	326 (6.3)	147 (6.7)	179 (6.0)	NS
Chronic kidney disease	13 (0.3)	7 (0.3)	6 (0.2)	NS
Cancer	11 (0.2)	5 (0.2)	6 (0.2)	NS
Hepatic disease	5 (0.1)	3 (0.1)	2 (0.1)	NS
Smoking	510 (9.8)	271 (12.3)	239 (8.0)	<0.001
Immunosuppression	10 (0.2)	2 (0.1)	8 (0.3)	NS
Asthma	184 (3.5)	65 (2.9)	119 (4.0)	0.046
HIV	6 (0.1)	6 (0.3)	0 (0.0)	0.006
Symptoms (n)		n (%)		
Populations manifesting symptoms (5190)	3,591 (70.2)	1,459 (66.3)	2,132 (71.5)	<0.001
Anosmia (4000)	266 (6.7)	92 (5.5)	174 (7.5)	0.01
Dysgeusia (4000)	291 (7.3)	106 (6.3)	185 (8.0)	0.041
Fever (5190)	1770 (34.1)	755 (34.3)	1,015 (34.0)	NS
Cough (5190)	2,183 (42.1)	853 (38.7)	1,330 (44.5)	<0.001
Nasal Congestion (5190)	1,018 (19.6)	402 (18.2)	616 (20.6)	0.034
Chest pain (5190)	595 (11.5)	218 (9.9)	377 (12.6)	0.002
Fatigue (5190)	1,359 (26.2)	549 (24.9)	810 (27.1)	NS
Difficulty breathing (5190)	403 (7.8)	151 (6.9)	252 (8.4)	0.035
Headache (5190)	2,421 (46.6)	951 (43.1)	1,470 (49.2)	<0.001
Chill (5190)	737 (14.2)	282 (12.8)	455 (15.2)	0.013
Myalgia (5190)	1,247 (24)	501 (22.7)	746 (25.0)	NS
Arthralgias (5190)	961 (18.5)	410 (18.6)	551 (18.5)	NS
Rhinorrhea (5190)	1,242 (23.9)	472 (21.4)	770 (25.8)	<0.001
Sore throat (5190)	2032 (39.2)	791 (35.9)	1,241 (41.6)	<0.001
Diarrhea (5190)	344 (6.6)	156 (7.1)	188 (6.3)	NS
Abdominal pain (5190)	184 (1.9)	67 (3.0)	117 (3.9)	NS

NS, Non-significant (*p* > 0.05). The chi-square test and U de Mann–Whitney were employed for statistical analysis.

### 3.2 Prevalence of respiratory viruses

**Figure 1:**
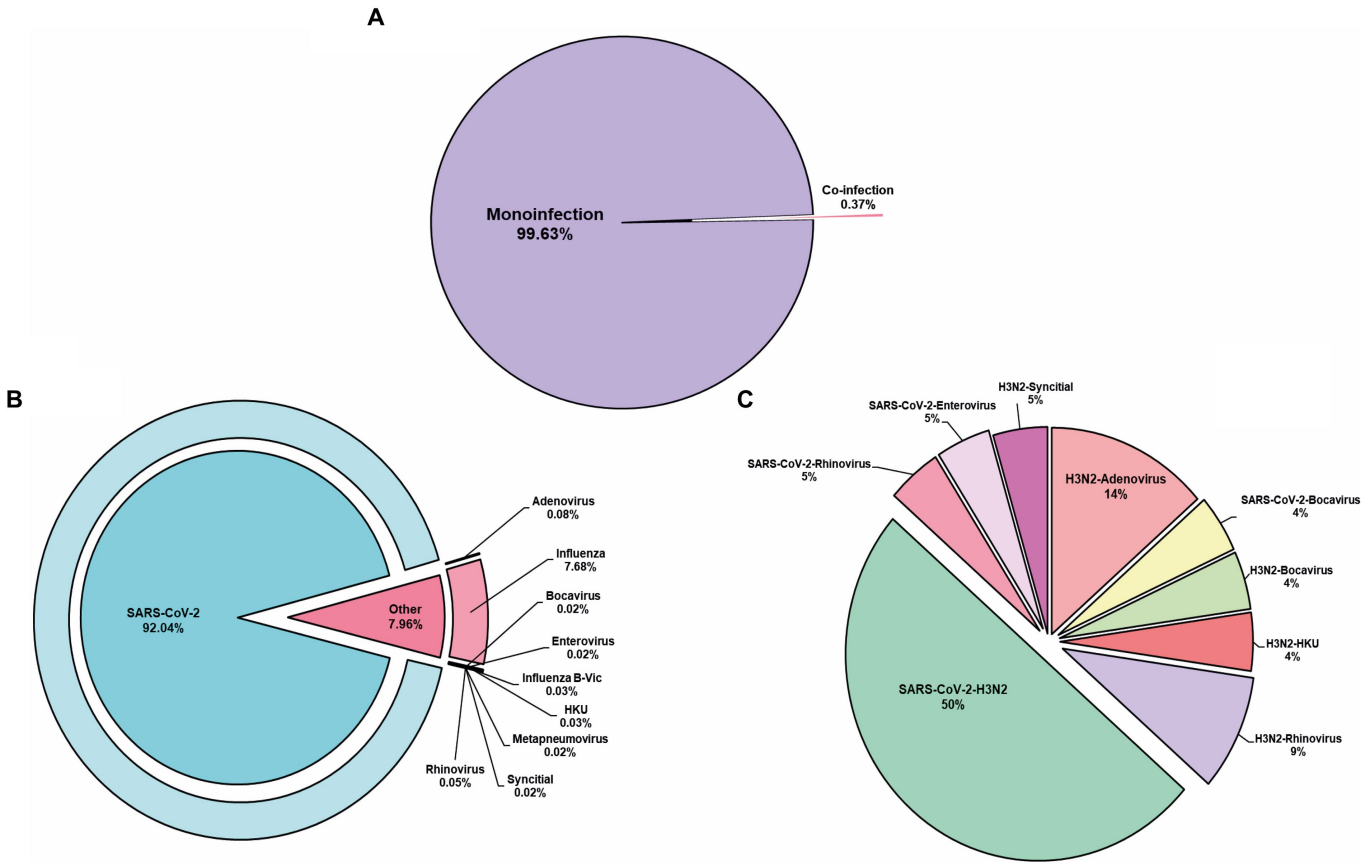
Respiratory viruses determined. **(A)** Mono-infection represents the major cause of disease found in our population. **(B)** From the mono-infection, SARS-CoV-2 was the prevalent virus identified. **(C)** Represents the co-infection identified. HKU, Human coronavirus.

From the analyzed samples, mono-infections were represented by 99.58% (*n* = 6,158), while the rest, 0.42% (*n* = 26), were coinfections (Figure 1A). SARS-CoV-2 was the most prevalent viral agent found as a mono-infection, with 5,703 individuals identified and a total of 481 cases of non-SARS-CoV-2 respiratory virus, representing 7.78% (Figure 1B). From this last, 479 participants were infected with influenza (477 corresponded to lineage A, from which 263 cases, 54.9%, were subsequently identified as H3N2 lineage), two of Influenza B (from the Victoria lineage), two human coronaviruses, five adenoviruses, one Bocavirus, three rhinoviruses, one Syncytial virus, and one Enterovirus (Figure 1B). The main coinfection observed was SARS-CoV-2 and Influenza A virus, detected in 13 samples. Besides SARS-CoV-2 and Influenza virus, HKU was the only one identified as a mono-infection. It is important to point out that influenza was present in most of the coinfection states. Likewise, coinfection cases involving SARS-CoV-2, Rhinovirus, and Enterovirus were identified (Figure 1C).

### 3.3 Impact of the social mobility in Jalisco and the reappearance of non-SARS-CoV-2 respiratory virus

**Figure 2:**
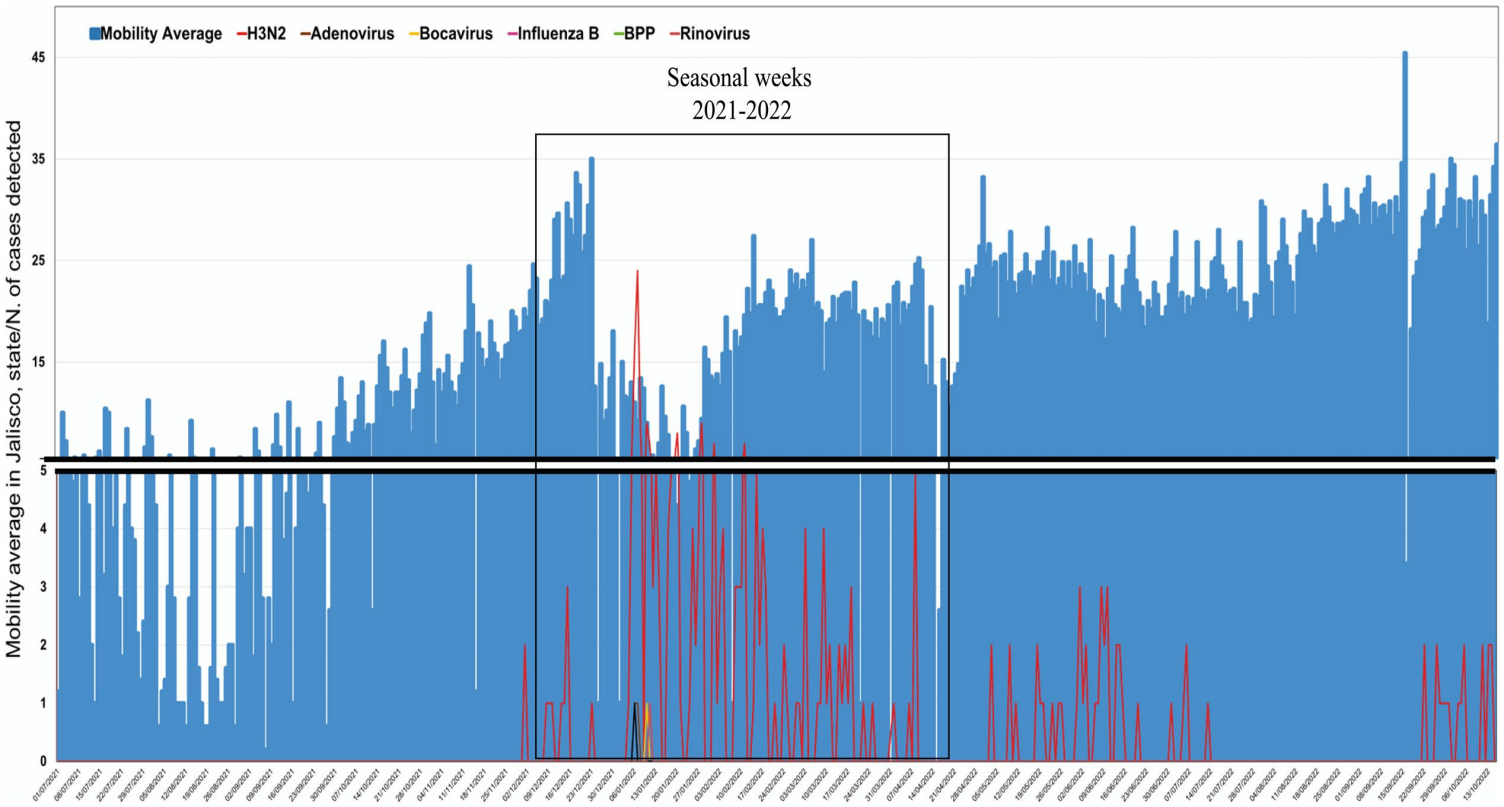
Impact of social mobility on viral respiratory detection. An increase in social mobility was observed in the second half of 2021, which correlates with the new viral respiratory detection.

According to the local public health institute database, no respiratory viruses were detected in West Mexico during the pandemic lockdown in 2020. Similarly, our epidemiological surveillance system has not detected the circulation of influenza in the region; nonetheless, influenza cases were reported at the end of 2021 (8). As so, we focused on evaluating if the reappearance of this virus was associated with the mobility change in Jalisco (Mexico) due to the removal of lockdown restrictions. The analysis showed that since December 2021, a re-emergence of Influenza infection cases was observed, in parallel with the trend of increased mobility observed at the end of October 2021 (Figure 2). The maximum peak of detected cases of respiratory viruses was registered during the fourth SARS-CoV-2 wave caused by the Omicron variant in January 2022, corresponding to the second epidemiological week. Additionally, we used the Kendall-tau correlation to quantify the relationship between the Influenza cases and the mobility data; mobility showed a positive correlation with infection cases of 0.214 (*p* < 0.001). No data further than October 2022 was retrieved due to the end of the aforementioned Google project.

### 3.4 Clinical impact of respiratory viruses

**Figure 3:**
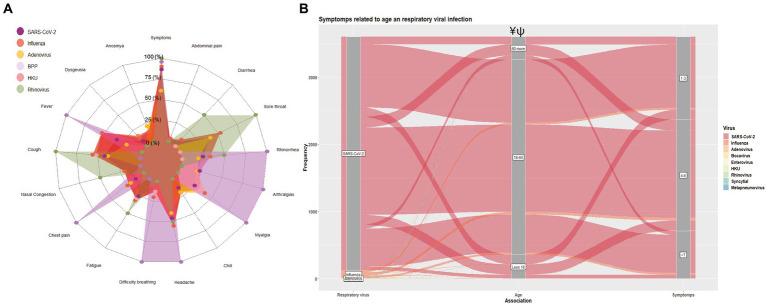
Impact of respiratory virus infection on the clinical presentation. **(A)** Prevalence of symptoms description according to the viral infection. **(B)** Sankey plot showing the distribution of the number of symptoms (0, 1–3, 4–6, and > 7) according to the age group and viral infection. Significant differences between age groups ¥: Influenza vs. SARS-CoV-2; ψ: Influenza/SARS-CoV-2 vs. Influenza. The chi-square test was used to compare proportions, and a value of *p* <0.05 was considered significant.

Illness produced by respiratory viruses presents a similar clinical behavior that can hardly be differentiated without a molecular diagnosis. In this regard, we recovered information such as symptoms and comorbidities (*n* = 5,841; missing clinical data of *n* = 343). Briefly, 3,591 individuals (69.3%) reported any symptoms; the female population exhibited a statistically significant increase compared to males (female = 59.4% vs. males = 40.6%; <0.001), with the highest proportion of people reporting 4–6 symptoms (*n* = 1,654; 46.06%). The people infected with the Influenza virus were prone to manifest illness-like symptoms at 90.37%; while Bocavirus, Human Coronavirus, and Rhinovirus had 100%, respectively; however, the information from Bocavirus, Human Coronavirus, and Rhinovirus should be interpreted cautiously since only a few cases were detected and mainly as a coinfection. In this sense, Influenza stands as an infection that causes an increased severity. On the other hand, 60.9% of subjects with SARS-CoV-2 presented symptoms (Figure 3A). Headaches (*n* = 2,421; 46.6%), cough (*n* = 2,183; 42.1%), sore throat (*n* = 2,032; 39.2%), and fever (*n* = 1,770; 34.1%) were the most frequent symptoms reported, independently of the viral infection.

Concerning viral infection and the related age (<18, 18–60, and > 60), we grouped the participants according to the number of symptoms reported (1–3, 4–6, and > 7) as is shown in Figure 3B; individuals infected with SARS-CoV-2 or Influenza in the age rank groups from 18 to 40 and 40 to 61 years old presented a significant highest proportion of symptoms (SARS-CoV-2: *n* = 2,905, 50.8%; 1,733, 30.3%; Influenza: 300, 12.2%, 92, 19.2%, respectively; *p* < 0.001): these population exhibited mostly 4–6 symptoms. This same age group showed the highest proportion of individuals with more than 7 symptoms (61.23 and 33.84, respectively). People from the 18–60 age group exhibited a higher proportion of symptomatology of different severity (80.6%), see Supplementary Table S1.

Regarding SARS-CoV-2 and Influenza coinfections, 100% (*n* = 9) manifested symptomatology; yet, when we evaluated this viral infection individually, influenza showed a significant difference (90.37%) compared to SARS-CoV-2 (60.9%; *p* < 0.001). Once that is re-grouped according to the number of symptoms, those infected with influenza tend to have a higher proportion of severe disease than SARS-CoV-2 since 26.4% of people had >7 symptoms. Notably, those co-infected with SARS-CoV-2 and Influenza were prone to have an augmented disease severity (33.3%; *p* < 0.001), represented by a higher number of reported symptoms. With respect to sex influence over the disease severity, females showed statistical differences versus males in symptomatology reported (females 59.4% vs. males 40.6%; <0.001); these differences were maintained in people with SARS-CoV-2 mono-infection. Regarding the population with Influenza infection, we only observed differences between sex group in those with >7 (females 33.8% vs. males 19.61%); see Table 2.

**Table 2:** The number of symptoms among respiratory viral infections as mono-infection and poli-infection.

Viral infection	Sex group	Symptomatic	Number of symptoms reported		
			1–3 n (%)	4–6 n (%)	>7 n (%)
Total (n, 5,841)	Total	3,591 (69.3)	1,230 (34.25)	1,654 (46.06)	707 (19.69)
	Male	1,459 (40.6)	532 (43.3)	633 (40.1)	264 (37.3)
	Female	2,132 (59.4)	698 (56.7)	991 (59.9)	443 (62.7)
	*p*-value	<0.001	<0.001	<0.001	<0.001
SARS-CoV-2 (n, 5,711)	Total	3,477 (60.9)	1,193 (34.3)	1,608 (46.3)	676 (19.4)
	Male	1,411 (40.6)	510 (36.2)	647 (45.8)	254 (18)
	Female	2066 (59.4)	683 (33.1)	961 (46.5)	422 (20.4)
	*p*-value	<0.001	<0.001	<0.001	<0.001
Influenza (n, 135)	Total	122 (90.4)	41 (33.6)	47 (38.5)	34 (27.9)
	Male	51 (41.8)	24 (47.06)	17 (33.33)	10 (19.61)
	Female	71 (58.2)	17 (23.9)	30 (42.3)	24 (33.8)
	*p*-value	NS	NS	NS	0.016
SARS-CoV-2 and Influenza (n, 9)	Total	9 (100)	4 (44.4)	2 (22.3)	3 (33.3)
	Male	3 (33.3)	2 (66.7)	1 (33.3)	0 (0)
	Female	6 (66.7)	2 (33.3)	1 (16.7)	3 (50)
	*p*-value	NS	NS	NS	NS
Influenza and Adenovirus (n, 3)	Total	3 (60)	1 (33.3)	1 (33.3)	1 (33.3)
	Male	1 (33.33)	0 (0)	0 (0)	1 (100)
	Female	2 (66.7)	1 (100)	1 (100)	0 (0)
	*p*-value	NS	NS	NS	NA
Influenza and Bocavirus (n, 1)	Total	1 (100)	0 ()	0 ()	1 (100)
	Male	1 (100)	0 (0)	0 (0)	1 (100)
	Female	0 (0)	0 (0)	0 (0)	0 (0)
	*p*-value	NA	NA	NA	NA
Influenza and Rhinovirus (n, 2)	Total	2 (100)	0 (0)	2 (100)	0 (0)
	Male	2 (100)	0 (0)	2 (100)	0 (0)
	Female	0 (0)	0 (0)	0 (0)	0 (0)
	*p*-value	NA	NA	NA	NA
Influenza B victoria (n, 1)	Total	1 (100)	0 (0)	0 (0)	1 (100)
	Male	0 (0)	0 (0)	0 (0)	0 (0)
	Female	1 (100)	0 (0)	0 (0)	1 (100)
	*p*-value	NA	NA	NA	NA
*p*-value on virus comparison		<0.001 ¥,ψ	<0.001 ¥	<0.001 ¥	<0.001 ¥

NS, non-significant; NA, non-aplicable. *P*-value < 0.001; ¥: Influenza vs SARS-CoV-2; ψ: Influenza/SARS-CoV-2 vs Influenza. The chi-square test and U de Mann–Whitney were employed for statistical analysis.

**Figure 4:**
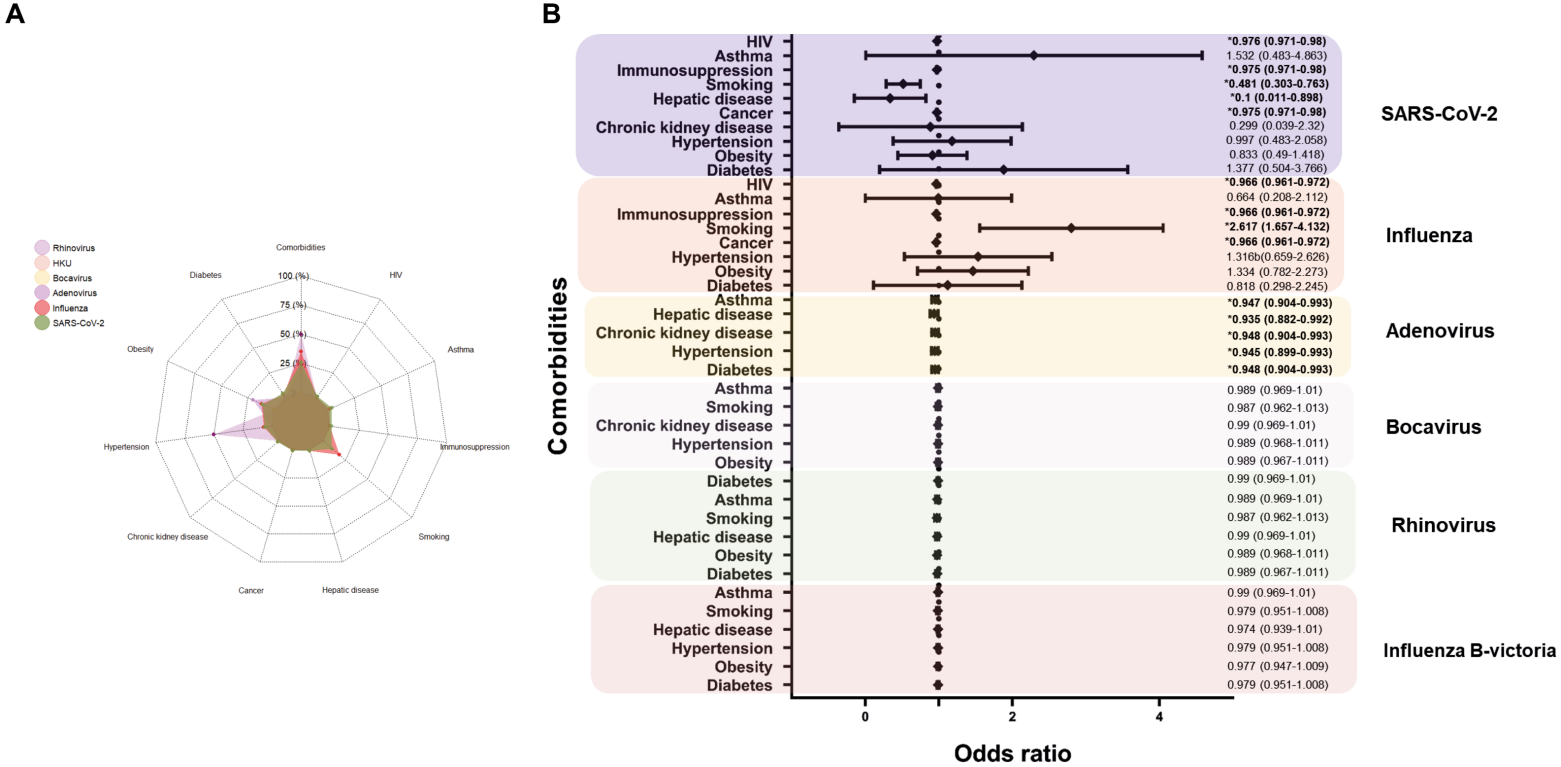
Association among respiratory viruses and comorbidities. **(A)** Prevalence of comorbidities description according to the viral infection. **(B)** Forest plot showing the risk for the viral infection according to the comorbidities; smoking showed a contrasting result as a risk factor for influenza and protection for SARS-CoV-2 infection.

In order to evaluate the impact of the comorbidities or unhealthy habits on respiratory viral infection, a univariate analysis was carried out. Firstly, we observed that individuals with rhinovirus have the largest proportion of comorbidities (50%), but since only two subjects responded to the survey, the data could not be representative. Patients monoinfected with Influenza and SARS-CoV-2 have 35 and 26% of previous comorbidities, respectively; the most reported was smoking (18 and 10%, respectively), followed by obesity (12 and 11%, respectively) and hypertension (7 and 6%, respectively; Figure 4A). Remarkably, in the context of Influenza infection, comorbidity represents a risk factor for the infection, with an OR = 1.86; IC 95%: 1.29–2.67, as smoking (OR = 2.62; IC 95%: 1.66–4.13). While these comorbidities are shown as a protective factor for SARS-CoV-2 infection (OR = 0.65; IC 95%: 0.45–0.94); in this sense, no risk factors were detected in SARS-CoV-2 diagnosed people, quite the opposite, cancer, hepatic disease, smoking, immunosuppression, and HIV were shown as a protective factor (Figure 4B). No risk factors were observed for adenovirus, but the number of cases was scarce.

### 3.5 Phylogenetic analysis of common respiratory viruses isolated in Mexico

A total of 334 respiratory viral sequences were found in Mexico (Table 3); 120 were partial sequences, and 214 were complete genomes; the most representative were influenza and rhinovirus, with a total of 123 sequences each, representing 36.82% of all viral respiratory infections reported from 2009 to 2018, (for influenza, mainly H1N1 were reported with a total of 114 sequences, and 9 were H3N2; while for rhinovirus serotype A was prevalent with 76 sequences, serotype B with 4, and serotype C a total of 43), followed by Bocavirus with a total of 37 sequences, with an 11.07% reported from 2001 to 2016, (all the sequences retrieved were HBoV1), Parainfluenza obtained a total of 27 sequences in the years 1995 to 2019, which represents 8.08% (the most significant were serotype 3 with 21 sequences and serotype 1 with six sequences). For the human syncytial virus, 21 sequences (6.28%) from 1980 to 2020 were recovered, mainly represented by serotype A with a total of 16 sequences and serotype B with 5. The metapneumovirus was less common, having only three genotype A sequences between 1983 and 2018 (0.89%) (Table 3). It is important to remark that for HKU, only one sequence has been sequenced and reported on public databases in Mexico, the OC63; no further sequences have been found. As for adenovirus, no sequences obtained for respiratory infections were reported (Supplementary Figure S1).

**Table 3:** Respiratory viruses reported in a public database from Mexico.

Respiratory virus	Sequences n (%)	Year
6	334	1980–2020
Influenza	123 (36.82)	2009–2018
H1N1	114 (92.86)	
H3N2	9 (7.31)	
Rhinovirus	123 (36.82)	2009–2018
Serotype A	76 (61.7)	
Serotype B	4 (3.25)	
Serotype C	43 (34.92)	
Bocavirus	37 (11.07)	2001–2016
Subtype HBoV1	37 (100)	
Parainfluenza	27 (8.08)	1995–2019
Serotype 1	6 (22.2)	
Serotype 3	21 (77.77)	
Syncytial	21 (6.28)	1980–2020
Serotype A	16 (76.19)	
Serotype B	5 (23.8)	
Metapneumovirus	3 (0.89)	1983–2018
Genotype A	3 (100)	

## 4 Discussion

In the last year, the world experienced a return to social activity, resulting from the removal of non-pharmacological interventions, which had been reported to have an impact on the reemergence of respiratory viruses since the WHO surveillance data reflected a substantial increase in global viral circulation, after the decrease during COVID-19 pandemic (16). Respiratory infections represent one of the leading causes of morbidity and mortality worldwide (17, 18). In México, the national health authorities reported 10 million respiratory infections in 2021, representing a decrease compared to 2019, which presented with more than 20 million infections (12). However, since the end of 2021 and the beginning of 2022, an increase in the number of cases of infection with non-SARS-CoV-2 respiratory virus was reported (9, 12), but the information is scarce; additionally, most of the efforts on respiratory virus diagnostics are focused solely on influenza (13), which affect the public health since the circulation of respiratory viruses different from Influenza and SARS-CoV-2 goes largely unnoticed. In the present document, we intend to describe the epidemiological and clinical characteristics of the respiratory viruses circulating in Jalisco, Mexico, and how social mobility might be involved in viral recirculation.

On average, the adult population experiences around two or five episodes of respiratory infection annually, with certain age groups, such as children and the older adult, more likely to be affected by more than seven events per year (7) demonstrated by numerous studies that prove pediatric populations are the most susceptible to respiratory infections (13, 19, 20), while the older adult are prone to demise (18). However, our study population was mostly 18 to 40 years old; this is in line with previous studies in the region of Jalisco, where people with SARS-CoV-2 were mainly in this age group (14, 21), probably due to the burden of economic activity placed on young adults. (22).

Throughout history, numerous infectious agents, including bacteria, viruses, and fungi, have been identified as causes of respiratory tract infections, with viral agents being the most prominent. Rhinovirus stands out as responsible for 50% of all respiratory infections worldwide, followed by human Coronaviruses (23). This information is consistent with Fernandes-Matano et al., which found rhinovirus as the most prevalent non-influenza respiratory virus circulating in Mexico, followed by syncytial and metapneumovirus (13). Historically, the influenza virus has been the main etiological agent of respiratory disease; because of this, the national health system has focused efforts on the diagnosis of this virus, ignoring the epidemiological distribution of other viral respiratory different to Influenza or SARS-CoV-2; therefore, the information regarding it needs to be improved. Herein, we reported that, in order, SARS-CoV-2, Influenza (lineage H3N2 and few cases of B Victoria), and adenovirus are the most prevalent, which is consistent with the epidemiological reports from the national health authority (12), with the exception of adenovirus, from which, the information in adults is limited and has been reported mainly in the pediatric population (24). Lately, public health institutions have been reporting a national prevalence of influenza H3N2 and Omicron subvariants (25).

Our data shows that social mobility was associated with the reemergence of the influenza virus at the end of 2021 and the beginning of 2022; additionally, new cases of other respiratory viruses were diagnosed during this period, although no correlation was observed between the incidence of these respiratory viruses and mobility. It has been well established that non-pharmacological interventions impact the number of infections reported; one of the most important measures is social mobility, which previously showed utility for this analysis (26–28). For the present study, no residential mobility data were included since we were looking for the impact of public spaces; Kishore K et al. demonstrated a negative correlation between the epidemiological data and the epidemiological indicators (28). Yet, it is important to remark that the correlation that we observed can be classified as weak; however, since LaDEER provides outpatient care for diagnosing respiratory viruses, the information may not reflect what occurs at the hospital level, where the correlation and number of infections may be even higher. Although mobility information from Google is no longer available, this was a handy tool that might have helped the national health authorities in the decision-making process during the COVID-19 pandemic; moreover, this and other mobility tools might be used to control future outbreaks, especially in Mexico, where monitoring of the epidemiological distribution of respiratory viruses is insufficient.

It has been published that respiratory disease symptoms share similar clinical characteristics with some clinical variations in the presence of certain symptoms (18). In order to differentiate the clinical presentation, a meta-analysis evaluating the clinical outcomes of the principal respiratory etiologies showed that respiratory diseases mainly present fever, sore throat, rhinorrhea, headache, myalgia, and cough; for COVID-19 and the common cold, fever was predominant, while influenza was characterized by myalgia and cold (29). A study published earlier in the COVID-19 pandemic reported that compared to influenza cases, runny nose, dyspnea, sore throat, and rhinorrhea were uncommon in patients with COVID-19 (30). These studies concord with our data since we reported that cough, sore throat, and fever were the most frequent symptoms manifested by our population; however, we observed that headache was the most recurrently described by the studied individuals but uncommon in the meta-analysis.

Recent studies focused on the population with COVID-19 from Jalisco showed that headaches were one of the most recurrent symptoms, which was in line with our findings (14, 21). Considering the coinfection, no statistical differences with mono-infections were found in our study; the information in this regard is inconsistent. A study published by Crotty et al. concluded that multiple viral infections were high-risk factors for patient mortality (31–33). Otherwise, several studies have shown that multiple viral infections do not increase the disease severity (31, 34–37); in fact, some studies have shown an inverse association among pediatric patients coinfected with respiratory viruses compared with sole infection (38, 39). This data is contradictory, but a possible explanation for a reduced risk of the worst clinical outcome could be due to secondary viral interference by the generation of interferon in infected patients because of a first viral entrance (40). It is difficult to solve this problem with our analysis since our study population is not significant. Future studies and meta-analyses that include SARS-CoV-2 and new influenza lineages should be conducted.

Our research shows a high risk of infection with influenza and smoking, contrary to the data from SARS-CoV-2 infected people, in which a low risk for viral infection was found. The unhealthy smoking habit and the risk for influenza infection association are well studied, and the data demonstrates that people who consume tobacco have more infections than those who do not (41). Nevertheless, regarding SARS-CoV-2 and smoking, the information is unclear. A similar result was found in California, where a significantly low-risk level for SARS-CoV-2 positivity was found (42, 43); Kashyap et al. suggest that a possible explanation could be a weak immune response and large and deep deliberate exhalations, which expel large quantities and concentrations of viral particles (44). Other possible mechanisms have been reviewed by Shariq-Usman et al., which include low production of pro-inflammatory cytokines, local vasodilatation, low expression of ACE2, and high production of nitric oxide; however, the authors recommend that this information should be taken with caution since several biases and knowledge gaps were identified (45), and it was demonstrated that smokers have a worst diseases prognosis at the time of infection (46, 47). Furthermore, when the data was evaluated according to the existence of any comorbidity, statistical analysis showed that people have lower odds of infection with SARS-CoV-2; this should be interpreted carefully since our data is from a local population, of which 80% present a comorbidity (48), leading to a bias in the calculation.

One of the principal limitations of this study is the lack of consideration for vaccination status against SARS-CoV-2 or other viruses. This is noteworthy because previous vaccination has been demonstrated to impact infection rates with various respiratory viruses, particularly the influence of the Influenza vaccine on SARS-CoV-2 infection. It’s crucial to acknowledge that there might be a statistical bias due to the small population size associated with viruses other than Influenza and SARS-CoV-2. Furthermore, this study provides data at the local level, and it’s essential to recognize that analyzing information at the local level can influence epidemiological statistics. Therefore, caution is advised when extrapolating these findings to estimate data at the state or national level. Lastly, it’s important to highlight that the diagnostic evaluation for respiratory viruses, aside from SARS-CoV-2 and Influenza, was limited. Consequently, the epidemiological burden information presented in this study may be underrepresented.

## 5 Conclusion

In conclusion, we present evidence regarding the social activation and the endemic respiratory virus reemergence as well the description of their prevalence in Jalisco, Mexico, which may lay the groundwork for follow-up in future studies that provide information in the establishment of prevention measures for epidemiological control. Nevertheless, many unanswered questions remain regarding the impact of reduced antigenic exposure to viral agents on the severity of respiratory infections in the coming years and its implications for rearranging the genotypic distribution of various respiratory viruses in the post-pandemic period.

## Data availability statement

The raw data supporting the conclusions of this article will be made available by the authors, without undue reservation.

## Ethics statement

The studies involving humans were approved by Comité de ética en Investigación, CUCS, Universidad de Guadalajara (registry number CONBIOÉTICA-14-CEI-002-20191003; protocol code: CI-04821). The studies were conducted in accordance with the local legislation and institutional requirements. The human samples used in this study were acquired from tacit consent through a privacy notice in the diagnosis results. This notice informed how patients could object to the processing of their data. Written informed consent for participation was not required from the participants or the participants' legal guardians/next of kin in accordance with the national legislation and institutional requirements.

## Author contributions

IP-U: Data curation, Methodology, Writing – original draft. NV: Funding acquisition, Project administration, Writing – review & editing. JM-V: Funding acquisition, Project administration, Writing – review & editing. MP-R: Methodology, Validation, Writing – review & editing. AC-A: Methodology, Validation, Writing – review & editing. RS-S: Methodology, Supervision, Writing – review & editing. AV-L: Methodology, Validation, Writing – review & editing. OG-G: Methodology, Validation, Writing – review & editing. JZ-M: Formal analysis, Writing – review & editing. MR-S: Methodology, Validation, Writing – review & editing. OV-S: Conceptualization, Project administration, Supervision, Writing – review & editing. MG-C: Conceptualization, Project administration, Supervision, Writing – review & editing.
