# Supplementary material for: Beyond SARS-CoV-2: epidemiological surveillance of respiratory viruses in Jalisco, Mexico
Source: Front Public Health. 2024 Jan 11;11:1292614. doi: 10.3389/fpubh.2023.1292614 (PMC10808461; doi:10.3389/fpubh.2023.1292614)

Supplementary Material

Supplementary Table 1. Disease severity related to age group.

	Age group			
	**Total**	**<18**	**18-60**	**>60**
	**n (%)**	**n (%)**	**n (%)**	**n (%)**
**Symptoms**	3591 (69.3)	355 (9.9)	2895 (80.6)	341 (9.5)
**1-3**	1230 (21)	145 (11.8)	966 (78.5)	119 (9.7)
**4-6**	1654 (28.3)	155 (9.4)	1336 (80.8)	163 (9.9)
**>7**	707 (12.1)	55 (7.8)	593 (83.9)	59 (8.3)

Supplementary Figure 1. Phylogenetic analysis of respiratory viruses identified in Mexico reported on public data-base. a) Influenza virus; H1N1 were prevalent. b) Parainfluenza virus; serotype 3 was prevalent. c) Metapneumovirus; only genotype A was found. d) Syncytial virus; serotype A was prevalent. e) Bocavirus; only serotype HBoV1 was reported. f) Rhinovirus; serotype A was prevalent in Mexico. The phylogenetic tree was constructed with the Neighbor-Joining algorithm, employing Tamura-Nei using bootstrap analysis of 10,000 replicates.

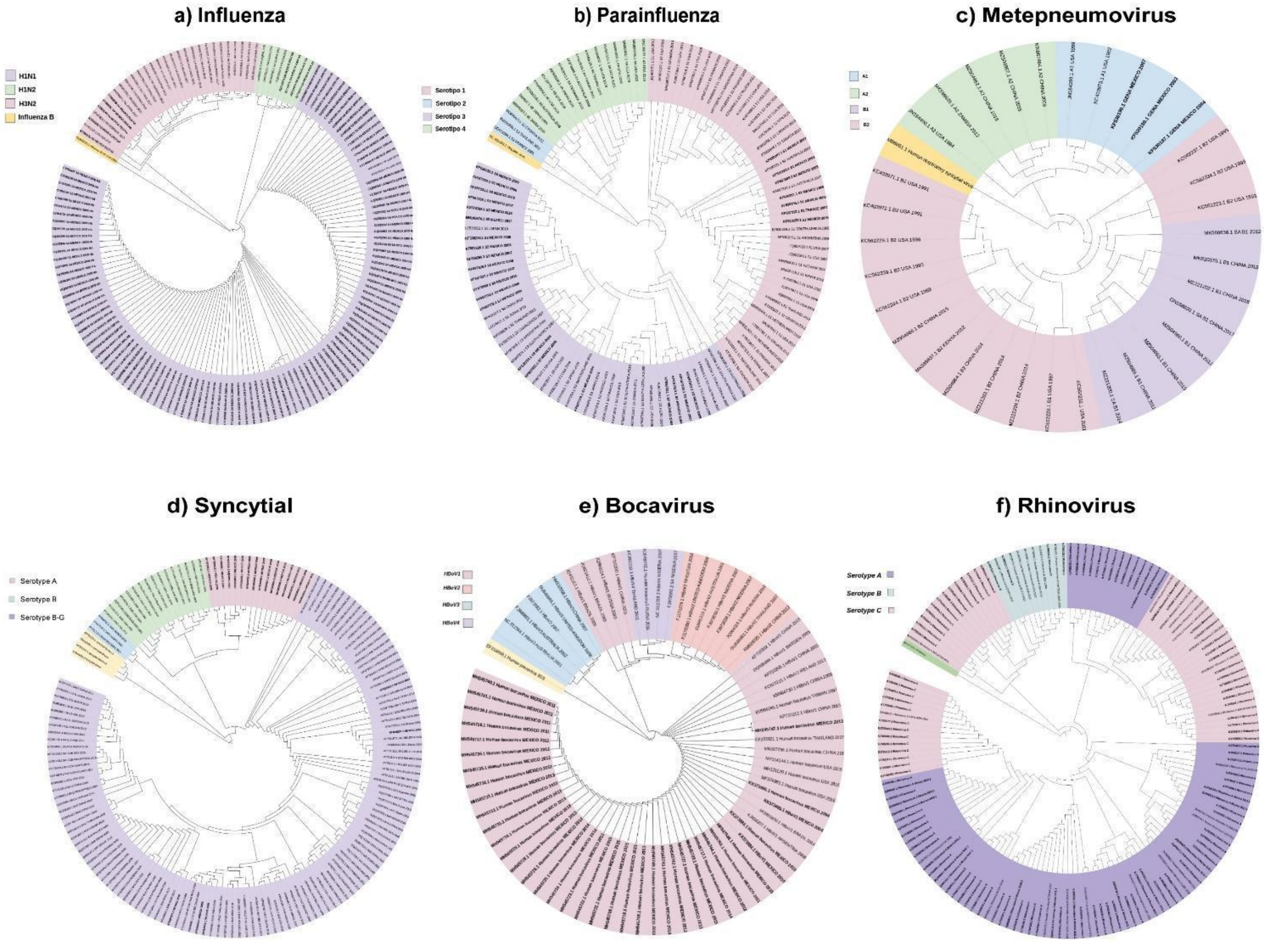

Supplement: Supplementary file 1 [file Data_Sheet_1.pdf]
